# Editorial: Sleep Spindles: Breaking the Methodological Wall

**DOI:** 10.3389/fnhum.2016.00672

**Published:** 2017-01-18

**Authors:** Christian O'Reilly, Simon C. Warby, Tore Nielsen

**Affiliations:** ^1^Blue Brain Project, École Polytechnique Fédérale de LausanneGeneva, Switzerland; ^2^Center for Advanced Research in Sleep Medicine, Centre de Recherche de l'Hôpital du Sacré-Cœur de MontréalMontreal, QC, Canada; ^3^Département de Psychiatrie, Université de MontréalMontreal, QC, Canada; ^4^Dream and Nightmare Laboratory, Center for Advanced Research in Sleep Medicine, Centre de Recherche de l'Hôpital du Sacré-Cœur de MontréalMontreal, QC, Canada

**Keywords:** sleep spindles, methods, sleep, open access

Research on sleep spindles and their correlates has progressed steadily over the last decade. The subject has evolved from a simple topic of investigation to an emerging research field, as indicated this year by the first international conference on sleep spindles in Budapest, Hungary, as well as the launching of a scientific journal (i.e., Sleep Spindles and Cortical Up States: A Multidisciplinary Journal) on this topic. This increasing interest has been fueled by reports of associations of sleep spindle characteristics with diseases such as schizophrenia (Ferrarelli et al., 2007, 2010; Manoach et al.), Parkinson's disease (Christensen et al.), REM sleep behavior disorder (Christensen et al., 2014; O'Reilly et al., 2015), Alzheimer's disease (Montplaisir et al., 1995; Rauchs et al., 2008), autism (Limoges et al., 2005), and mental retardation (Shibagaki et al., 1982), with recovery processes following brain stroke (Gottselig et al., 2002), with cognitive faculties such as memory consolidation and intelligence (Fogel and Smith, 2011), and with sleep preservation (Landis et al., 2004; Dang-Vu et al., 2010; Schabus et al., 2012). Nonetheless, many methodological difficulties have been encountered in reliably detecting sleep spindles. Hence, this research topic was launched as a forum for proposing better practices in the study of sleep spindles and to provide new insights on spindle correlates. Authors were invited particularly to propose open-access resources that could help promote improved methods and support standardization in the field.

## Contributions

A total of 17 papers were accepted for publication on the research topic, with 10 being focussed particularly on methodological issues such as spindle detection and the remaining seven providing new insights on sleep spindle correlates.

### Methodological advances

Different approaches were investigated for tackling the difficult task of detecting sleep spindles automatically, including the use of continuous wavelet transform (Adamczyk et al.; Tsanas and Clifford), complex demodulation (Ray et al.), matching pursuit (Durka et al.), and morphological component analysis of a sparse representation of EEG segments using the discrete tunable Q-factor wavelet transform (Lajnef et al.).

Among the developments proposed for sleep spindle detection, some concentrate on particular issues associated with clinical applications or with better control of factors impacting spindle variability. For clinical applications, Tsanas and Clifford propose a detector deployable with single-lead recordings that does not require prior sleep stage scoring, two arguably important features for daily clinical use. From the perspective of better controlling factors impacting on the variability of spindle properties, Ray et al. propose an algorithm accounting for variability across the night, across derivations, and across subjects while keeping the number of user-defined parameters to a minimum. Ujma et al. propose arguments that support dynamically determining, for each subject, the threshold used for separating fast from slow spindles according to the spectral structure of the individual's EEG. Such individually defined thresholds are used in the detector proposed by Adamczyk et al. Some of the proposed detection techniques also aim at a more general detection framework, which could manage a larger set of sleep waveforms, e.g., including not only sleep spindles but also K-complexes (Durka et al.; Lajnef et al.).

In their contribution to the special issue, O'Reilly and Nielsen suggest modified versions of four standard detection algorithms to improve temporal resolution in determining spindling time windows. They also provide an in-depth analysis of the limitations and pitfalls associated with spindle detection assessment. Pitfalls and guidelines for spindle detection can also be found in an opinion paper by Ktonas and Ventouras.

O'Reilly et al. take a different approach and propose a semi-automated detector relying on machine learning. In this approach, sigma-band amplitude, and spectral ratio features are used in a first step followed by hierarchical clustering based on frequency and spatial position of the spindle along the anterior–posterior axis of the scalp, so as to capture differences between classes of slow and fast spindles. This proposal falls to some extent at the opposite end of a spectrum when compared to the proposal of Tsanas and Clifford; whereas the former tries to benefit from high-density grid recordings for research purposes, the latter focuses on obtaining reliable detections from minimal information for clinical uses. Related to the context of the former study are the comments from the Ktonas and Ventouras opinion paper on the estimation of intracranial current sources of sleep spindles, a topic that is likely to become increasingly important with the improvement of source localization algorithms, and the wider spread of EEG high-density sensor grids.

Targeted more toward developing an improved representation of sleep spindles than toward detection *per se*, Palliyali et al. propose to parameterize the structure of spindles using a quadratic parameter sinusoid. In their study, they provide a detailed analysis of the parameters' sensitivity and show, among other findings, that these parameters take distinct values for spindle vs. non-spindle epochs.

More closely related to the very definition of sleep spindles, Nader and Smith propose some controversial results that challenge the traditional view of sleep spindles by investigating sleep spindles in atypical stages (e.g., REM) and frequency bands (e.g., 16–18.5 Hz).

It is noteworthy that a significant number of contributed papers (Durka et al.; O'Reilly and Nielsen; Palliyali et al.; Tsanas and Clifford) include an evaluation of their detection algorithms on a common database (the second subset of the Montreal Archive of Sleep Studies; O'Reilly et al., 2014), thereby providing much better cross-study comparisons than if they had been evaluated using different expert scorings (O'Reilly and Nielsen).

### Proposal of open-access tools

A valuable outcome of this research topic is the release of many open-access resources for studying sleep spindles. This is the case for the matching pursuit detector of Durka et al. which is provided as part of the Signal Viewer, Analyzer, and Recorder On GPL (SVAROG) package available at http://braintech.pl/svarog; of the detectors evaluated in O'Reilly and Nielsen which are part of the open-source Python package Spyndle available at https://bitbucket.org/christian_oreilly/spyndle; and of the single-lead detector of Tsanas and Clifford available as a Matlab source code at https://people.maths.ox.ac.uk/tsanas/. Similarly, some other Matlab packages are available directly from the authors Adamczyk et al., Lajnef et al., and Ray et al. Finally, the detector from O'Reilly et al. has been implemented as a Brainstorm (Matlab) process for easy integration with neuroimaging pipelines implemented in this environment. It is also available from the authors.

### Other advances in the study of sleep spindling

Although primarily targeted at discussing methodological issues related to the investigation of sleep spindles, other types of validational studies of sleep spindles were included to broaden the scope of this research topic. This includes two papers on the relationship between sleep spindles and mental faculties in adolescents, one examining how spindling frequency is related to processing speed as well as the relationship between performance on a motor task and sleep quality (Nader and Smith), the other assessing links between sleep spindles and fluid IQ, with a particular attention to sex as a modulating factor (Bódizs et al.). Similarly, Astill et al. studied links between performance on a motor task and sleep spindling in children; they found better performance with faster EEG, in accordance with what was reported for adolescents (Nader and Smith).

Two contributions examine how diseases are correlated with properties of sleep spindles, one focusing on Parkinson's disease (Christensen et al.), the other on schizophrenia (Manoach et al.). Others report correlates of sleep spindles including age-related impact of sleep-deprivation (Rosinvil et al.) and level of insomnia symptoms in response to a stressful situation (Dang-Vu et al.). Finally, Adamczyk et al. report on the influence of genetics on the variability of slow and fast sleep spindles.

These studies demonstrate once more that sleep spindling is an important physiological process that can be modulated by many conditions. They also further highlight the relevance of establishing the role of sleep spindles in the normal functioning of the brain.

## Conclusion

With the publication of an e-book compiling all these contributions on sleep spindle correlates and methodological advancements for their study, another step has been taken in advancing the foundations of this emerging research field. It is the hope of its editors that these papers will support the continued enhancement of methods used to study sleep spindling, promote the establishment of commonly used open-access research tools and, eventually, foster a better understanding of the mechanisms involved in sleep spindles and their role in neurophysiological and pathological processes.

## Author contributions

COR wrote the first draft. All authors revised and edited the manuscript.

## Conflict of interest statement

The authors declare that the research was conducted in the absence of any commercial or financial relationships that could be construed as a potential conflict of interest.
